# Comprehensive Analysis of Glutamate Receptor-like Genes in Rice (*Oryza sativa* L.): Genome-Wide Identification, Characteristics, Evolution, Chromatin Accessibility, gcHap Diversity, Population Variation and Expression Analysis

**DOI:** 10.3390/cimb44120437

**Published:** 2022-12-16

**Authors:** Yingyao Shi, Wei Zeng, Minhui Xu, Hua Li, Fanlin Zhang, Zulong Chen, Gatera Anicet, Shiji Huang, Yuheng Huang, Xiyu Wang, Junhao Li, Xinyu Zhang, Yuxing Zheng, Shamsur Rehman

**Affiliations:** 1 College of Agronomy, Anhui Agricultural University, Hefei 230036, China; 2 Innovation Center for Sustainable Forestry in Southern China, Key Laboratory of Forest Genetics & Biotechnology, College of Biology and the Environment, Nanjing Forestry University, Nanjing 210037 China

**Keywords:** glutamate receptor-like genes, evolution, expression variation, chromatin accessibility, gcHap, population variation

## Abstract

Glutamate receptors (GLR) are widely present in animals and plants, playing essential roles in regulating plant growth, development and stress response. At present, most studies of GLRs in plants are focused on *Arabidopsis thaliana*, while there have been few studies on rice. In this study, we identified 26 *OsGLR* genes in rice (*Oryza sativa* L.). Then, we analyzed the chromosomal location, physical and chemical properties, subcellular location, transmembrane (TM) helices, signal peptides, three-dimensional (3D) structure, cis-acting elements, evolution, chromatin accessibility, population variation, gene-coding sequence haplotype (gcHap) and gene expression under multiple abiotic stress and hormone treatments. The results showed that out of the 26 *OsGLR* genes, ten genes had the TM domain, signal peptides and similar 3D structures. Most *OsGLRs* exhibited high tissue specificity in expression under drought stress. In addition, several *OsGLR* genes were specifically responsive to certain hormones. The favorable gcHap of many *OsGLR* genes in modern varieties showed obvious differentiation between *Xian/indica* and *Geng/japonica* subspecies. This study, for the first time, comprehensively analyzes the *OsGLR* genes in rice, and provides an important reference for further research on their molecular function.

## 1. Introduction

In 280 BC, Aristotle asserted that plants also have minds and can perceive the world as animals do. Recent studies have demonstrated that plants can indeed use their “wisdom” to adapt to changes in the external environment. With the rapid development of electrophysiology as well as cell and molecular biology, numerous molecules in plants have been found to perform neurological-like functions, which paves the way to explore the mystery of “plant neurology”. Glutamate acts as an excitatory neurotransmitter in the vertebrate central nervous system, facilitating long-range information exchange via activation of glutamate receptor channels [1]. *Glutamate receptor-like* (*GLR*) genes are widely found in animals and plants. There are various *GLR* genes in animal neurons and glial cells, which can be mainly divided into ionotropic glutamate receptors and metabotropic glutamate receptors.

Ionotropic glutamate receptors (iGluRs), such as kainate, AMPA and NMDA receptors, are glutamate-gated ion channels mainly expressed in the brain [2]. The iGluRs are complete membrane proteins consisting of four large subunits (>900 residues), including the extracellular amino-terminal domain (ATD) with a signal peptide, the extracellular ligand-binding domain (LBD), the transmembrane domain (TMD) with M1-M4 helixes, and the intracellular carboxyl-terminal domain (CTD), which form a central ion channel hole [2]. Plant glutamate receptor-like (GLRs) are specific membrane proteins with ligand-gated ion channel activities. It has been demonstrated that plant GLRs have a high degree of homology with non-NMDA receptors [3]. Fully functional plant GLRs have similar structures to iGluRs in animals, which also consist of four subunits [4]. Since it is difficult to study these GLRs in humans due to the complexity and ethical concerns, studies of them in plants may facilitate a better understanding of the more complex functions such as memory, learning or neurodegenerative diseases from the perspective of fundamental cellular biology.

Rice (*Oryza sativa* L.) is an important model species for monocot plants and cereals, such as maize (*Zea mays*), wheat (*Triticum aestivum* L.), barley (*Hordeum vulgare* L.) and sorghum (*Sorghum bicolor*). Therefore, it is of great significance to carry out basic research on *GLR* genes in rice. However, there have been few studies of GLR-related studies in rice. Li et al. [5], for the first time, screened an *osglr3.1* mutant from a rice T-DNA insertion library, and *osglr3.1* was found to be critical for the division and survival of individual cells in root apical meristems. In 2016, Ni et al. [6] identified 13 *OsGLR* genes solely through the Phytozome website (http://www.phytozome.net/, accessed on 29 July 2022). In this study, we identified 26 *OsGLR* genes in *Oryza sativa* by BLAST, HMM search and literature analysis [5,6,7], which genes were then divided into four subgroups. Subsequently, a comprehensive analysis of *OsGLR* genes was carried out, including the chromosomal location, physical and chemical properties, subcellular location, phylogenetic relationship, conserved motif, transmembrane (TM) helices, signal peptides, three-dimensional (3D) structure, cis-acting elements, evolution, chromatin accessibility, population variation, gene-coding sequence haplotype (gcHap) and gene expression analysis under multiple abiotic stress and hormone treatments. The data provide an important reference for further exploring the molecular functions of *OsGLR* genes in regulating rice growth and stress response.

## 2. Materials and Methods

### 2.1. Identification and Chromosomal Location of OsGLR Genes

To identify all GLR genes in the whole genome of rice, we first downloaded the whole genome data of rice (*Oryza sativa Geng/japonica*), tomato (*Solanum lycopersicum*) and *Arabidopsis thaliana* from the Ensembl plant database (http://plants.ensembl.org/index.html, accessed on 29 July 2021), and constructed a local database with protein sequence files. Then, we downloaded the GLR protein (ID: PF00060), searched PF00060 with hidden Markov models (HMM) from the Pfam database (http://pfam.xfam.org/, accessed on 29 July 2021) and used the HMM-search program of the HMMER software package (European Bioinformatics Institute, Cambridge, UK) to screen protein sequences containing this domain in rice. By using Pfam, SMART (http://smart.emblheidelberg.de/, accessed on 29 July 2021) and the NCBI-CDD database (https://www.ncbi.nlm.nih.gov/structure/cdd/cdd.shtml, accessed on 29 July 2021) to detect candidate proteins, and after removal of protein sequences containing incomplete domains, and analysis of the GLR-related literature, we finally identified all *OsGLR* genes [6], renaming them according to their positions on the chromosome.

The chromosomal location of the *OsGLR* genes was determined through the online website (http://mg2c.iask.in/mg2c_v2.1/, accessed on 22 July 2021). According to the website instructions, the information for all the 26 OsGLRs was sorted into two input data sets: input1 (gene location, including 1. gene_id; 2. gene_start; 3. gene_end; 4. chr_id; 5. gene_color) and input2 (chromosome length, including 1. chr_id and 2.chr_length).

### 2.2. Structural Characteristics and Phylogenetic Analysis of OsGLR Genes

We first used the software TBtoolsv0.66833 (South China Agricultural University, Guangzhou, China) to extract the protein sequences of the *OsGLR* genes from the rice genome data [8], and we predicted the physical and chemical properties of all OsGLRs on the website Expasy (https://web.expasy.org/protparam/, accessed on 22 July 2021). After extracting the position of the UTRs, introns, and exons of OsGLRs from the RAP-BD (the Rice Annotation Project Database) website, the structural information for OsGLRs was obtained and visualized in GSDS [9] (http://gsds.gaolab.org/, accessed on 22 July 2021). WoLF PSORT can predict the subcellular localization of proteins based on their amino acid sequences. Therefore, the subcellular localization of all *OsGLR* genes was completed on the online website (https://wolfpsort.hgc.jp/, accessed on 22 July 2021). For phylogenetic tree analysis, we downloaded the genome sequences of GLRs in rice (*Oryza sativa* Japonica Group), tomato (*Solanum lycopersicum*), and *A. thaliana* from the Ensembl plant database (http://plants.ensembl.org/index.html, accessed on 22 July 2021). MEGA [10] (Molecular Evolutionary Genetics Analysis,) is a very powerful software for molecular evolutionary genetic analysis, which can be used for sequence alignment, evolutionary tree inference, estimation of the molecular evolution rate and validation of evolutionary hypotheses. The phylogenetic tree of OsGLR, SlGLR and AtGLR sequences was constructed using the ML (maximum likelihood) method in MEGA7, and beautified on the online website (iTOL https://itol.embl.de/, accessed on 22 July 2021).

### 2.3. Prediction of Transmembrane Helices and Signal Peptide of the OsGLR Genes

Prediction of transmembrane (TM) helices was performed with reference to the guide for the online website TMHMM Server v.2.0 (https://services.healthtech.dtu.dk/service.php?TMHMM2.0, accessed on 23 July 2021). Protein sequences of the 26 OsGLRs were converted into FASTA format and submitted using the default parameters on the online website. The SignalP 5.0 server can predict the presence of signal peptides and the locations of their cleavage sites in proteins [11]; it is based on a deep convolutional and recurrent neural network architecture including a conditional random field. The deep recurrent neural network architecture is more powerful in recognizing sequence motifs with varying lengths, such as signal peptides, than traditional feedforward neural networks. The prediction method was similar to TM prediction. Protein sequences of the 26 OsGLRs were converted into FASTA format and input into the online website (https://services.healthtech.dtu.dk/service.php?SignalP5.0, accessed on 23 July 2021). Eukarya was selected as the Organism Group and the other parameters were the default values.

### 2.4. Three-Dimensional (3D) Structure and Conserved Motifs of OsGLR Genes

The 3D structure can provide a wealth of information on the biological function and evolutionary history of macromolecules. It can be used to examine the relationship between the sequence structure and function, protein interactions and active sites. The protein sequence of each OsGLR was used to predict their 3D structure with the Phyre2 (Structural Bioinformatics Group, Imperial College, London, UK) [12] (Protein Homology/analogY Recognition Engine V 2.0) online website (http://www.sbg.bio.ic.ac.uk/phyre2/html/page.cgi?id=index, accessed on 16 July 2021). The pipeline mainly includes the following steps: 1. detection of sequence homologs with PSIBlast; 2. prediction of secondary structure and disorder with Psipred and Disopred; 3. construction of an HMM of the sequence based on the homologs detected above; 4. scanning of this HMM against a weekly updated library of HMMs of proteins with experimentally solved structures; 5. construction of 3D models of the protein based on the alignment between the HMM of the sequence and the HMMs with known structures; 6. modelling of insertions and deletions using a loop library, a fitting procedure (cyclic coordinate descent) and a set of empirical energy terms; 7. modelling of amino acid side chains using a rotamer library from Roland Dunbrack’s laboratory and our own implementation of a fast graph-based approach (R3) to optimize the choice of rotamer for each sidechain whilst avoiding steric clashes; 8. submission of the top model (if sufficiently confident) for binding site prediction by 3DLigandSite(Structural Bioinformatics Group, Imperial College, London, UK); 9. prediction of transmembrane helix and topology by memsatsvm. Finally, the predicted 3D structure results were visualized in PyMOL 4.5.0 software (Warren Lyford DeLano, Philadelphia, PA, USA) [13]. MEME (Multiple Em for Motif Elicitation) (National Institutes of Health, Bethesda, MD, USA) can discover novel, ungapped motifs (recurring, fixed length patterns) and split variable-length patterns into two or more separate motifs [14]. For the motif distribution in OsGLR sequences, we selected Zero or One Occurrence per Sequence (zoops) and 10 conserved motifs. All other parameters were set as their default values (https://memesuite.org/meme/tools/meme, accessed on 16 July 2021).

### 2.5. Cis-Acting Regulatory Elements, Functional Interaction Network and Chromatin Accessibility of OsGLR Genes

Prediction of cis-acting regulatory sites was carried out through the online website (http://bioinformatics.psb.ugent.be/webtools/plantcare/html/, Last accessed on 26 July 2021). The whole genome sequence of rice was used to extract the 2000 bp sequence before the target gene promoter in TB tools v0.66833 Toolkit [8] (South China Agricultural University, Guangzhou, China). Four genes were not in the annotation file, which were downloaded separately from the Ensembl Plant Database. On the PlantCARE (http://bioinformatics.psb.ugent.be/webtools/plantcare/html/, accessed on 26 July 2021) website, the cis-acting regulatory elements of OsGLRs were predicted, and the results were visualized in Gene Structure Display Server (GSDS) [9] (http://gsds.gaolab.org/, accessed on 26 July 2021). The functional interaction network analysis was completed according to the instructions on the online website (https://www.stringdb.org/, accessed on 26 July 2021). STRING used a spring model to generate the network images. Nodes were modeled as masses and edges as springs. The final positions of the nodes in the image were computed by minimizing the ‘energy’ of the system. The amino acid sequences of the 26 OsGLRs were used to predict the functional interaction protein on the website STRING (https://www.stringdb.org/, accessed on 26 July 2021). Prediction of variant effects of chromatin accessibility was carried out through the online website (http://ricevarmap.ncpgr.cn/effect_predict/, accessed on 26 July 2021). RiceVarMap v2.0 [15] (Chinese Academy of Agricultural Sciences, Peking, China) is a comprehensive database for rice genomic variation and its functional annotation, providing curated information for 17,397,026 genomic variations (including 14,541,446 SNPs and 2,855,580 small INDELs) from the sequencing data of 4726 rice accessions. For chromatin accessibility, we collected six tissues (root (RT), young leaf (YL), flag leaf (FL), young panicle (YP), lemma and palea (LP), and stamen and pistil (SP)) of Zhenshan 97 (a *Xian/indica* variety) for the ATACseq experiment, with at least two replicates for each tissue. After mapping to the reference genome, an average of 39.9 million qualified fragments were obtained per sample. The chromatin accessibility results of OsGLRs were downloaded and plotted.

### 2.6. Population Variation, Gene-Coding Sequence Haplotype (gcHap) and KAKS of OsGLR Genes

Population variation analysis was carried out based on resequencing of 3010 rice accessions [16], which were divided into five subspecies, including GJ (*Geng/japonica*, 801 accessions), XI (*Xian/indica*, 1764 accessions), AUS (aus/boro, 221 accessions), ARO (aromatic basmati/sadri, 101 accessions) and ADM (admixed, 123 accessions). All these five subspecies were further grouped into 12 groups according to the classification of their corresponding rice accessions. These groups included five subgroups (XI1–5) of the XI subspecies AUSG6, four subgroups (GJ7–10) of the GJ subspecies AROG11, and admixtures (ADM). All genes were categorized according to their presence/absence in the 453 high-quality accessions. Core genes were defined as genes existing in all high-quality rice accessions. Distributed genes were defined as genes existing in significantly less than 99% of accessions (binomial tests, *p*-value < 0.05, null hypothesis is “loss rate < 1%”). Candidate core genes were taken as those existing in > 99% (not all) of high-quality rice accessions (binomial test, fdr < 0.05). Random genes were defined as genes without differences among groups and subgroups.

By using the identified OsGLRs for gene distribution and presence frequency analysis, population distribution results were obtained from the full dataset of 32 million Nipponbare-based SNPs from 3010 rice accessions (http://cgm.sjtu.edu.cn/3kricedb/search.php, accessed on 26 April 2021). Haplotype information [17] was obtained from the 3K database website (https://www.rmbreeding.cn/Index/, accessed on 26 April 2021), and modern varieties were defined in the literature [18]. Each gcHap of the *OsGLRs* genes was constructed by the concatenating SNPs in the CDS region, in which synonymous SNPs were ignored. KAKS was calculated in TBtools Toolkit (South China Agricultural University, Guangzhou, China) using the protein sequence and CDS sequence of *OsGLRs*, and the divergence time (Mya) was calculated using the formula: Mya = Ks/2λ × 10^−6^, where λ = 6.5 × 10^−9^.

### 2.7. Expression Difference of OsGLR Genes under Different Hormone Treatments

Gene expression data generated by the Affymetrix ATH1 (Thermo Fisher Scientific, Massachusetts, USA) array were normalized by the GCOS method, with a trimmed mean target intensity (TGT) value of 100. Most tissues were sampled in triplicate. The tissue expression data of all the *OsGLR* genes were downloaded from the BAR (The Bio-Analytic Resource for Plant Biology) database (http://www.bar.utoronto.ca/, accessed on 8 October 2021), and processed and visualized in TBtools Toolkit using the default settings. Gene expression data under abiotic stress conditions were obtained from http://rice.uga.edu/expression.shtml, accessed on 8 October 2021. The values of presence/absence variation were assigned for digital gene expression (DGE) libraries, and genes were regarded as ‘expressed’ if at least one sequence read was mapped uniquely within an exon.

The seeds of rice (Nipponbare) were sterilized with 70% ETOH and 1% hypochlorous acid. After disinfection, the seeds were imbibed and germinated in distilled water. The germinated seeds were transplanted into a 96-well plate and grown in a growth chamber at 28 °C, 24 h of light and 60% humidity. After three days of germination, the seedlings were transferred to the medium diluted five times with Yoshida nutrient solution [19] and adjusted to pH 5.5 with 1 mMMES + 1 M NaOH. The seedlings were allowed to grow for another four days. The medium was updated every two days. The 7-day-old seedlings were transferred to a medium containing plant hormones (50 μM ABA + 0.1% Ethanol, 10 μM GA + 0.1% Ethanol, 10 μM IAA + 0.1% Ethanol, 1 μM BR + 0.1% Ethanol, 1 μM CK + 0.02% DMSO and 100 μM JA + 0.02% DMSO). Shoot samples were collected at 0, 1, 3, 6 and 12 h after hormone treatment. Root samples were collected at 0 min, 15 min, 30 min, 1 h, 3 h and 6 h after hormone treatment. TRIzol (TIANGEN, W9330, Peking, China) was used to extract RNA from a total of 138 samples, and a Takara reverse transcription kit (SparkJade, AG0304, Shandong, China) was used to reverse message RNA into cDNA, which was diluted by five times and amplified by PCR MIX (SparkJade, SMQFK, Shandong, China). These RNA were labeled with Cy3 (mock treatment) and Cy5 (hormone treatment), and used for hybridization using the Agilent two-color microarray analysis system. The time-course expression profile for each gene is shown as the log-ratio of the signal intensity (log2 Cy5/Cy3). 

### 2.8. Semi-Quantitative PCR Analysis of OsGLR Genes under Low-Temperature Stress

The dormancy of Nipponbare (*Oryza sativa* L. *japonica*) seeds was broken in a drying oven (Boxun Ltd., GZX9240MBE, Hefei, China) at 43 °C for 5 days, and then 250 round and plump seeds of Nipponbare were selected and disinfected with 5% hypochlorous acid for 1 h. After disinfection, the sample was rinsed with ddH_2_O four to five times, and then put in 10 × 9 cm glass petri dishes with two layers of germination paper, followed by the addition of 10 mL distilled water for imbibing and germination. The germinated rice seeds were transplanted into a 12 × 8 cm germination box containing Hoagland nutrient solution. The nutrient solution was changed every four days, and cultivation was carried out under natural conditions. When the seedlings are cultivated to the three-leaf stage, low-temperature (4 °C) stress treatment was performed. Leaf samples were collected in liquid nitrogen at 0 h and 3 h, 6 h, 9 h, 12 h, 24 h and 48 h after low-temperature stress treatment, with sampling of seven seedlings at a time, and the experiment was repeated three times. The collected rice leaf samples were stored in a refrigerator (Haier, DW86L486, Qingdao, China) at –80 °C for later use. The primers of *OsGLR* genes are shown in Table 1. TRIzol (TIANGEN, W9330, Beijing, China) was used to extract RNA from rice leaf tissue, and a Takara reverse transcription kit (SparkJade, AG0304, Shandong, China) was used to reverse message RNA into cDNA, which was diluted five times and amplified by PCR MIX (SparkJade, SMQFK, Shandong, China).

## 3. Results

### 3.1. Genome-Wide Identification and Characterization of the OsGLR Genes in Rice

The *OsGLR* genes in the whole genome of *Oryza sativa* were identified with BLAST, HMM search and literature analysis [5,6]. Finally, a total of 26 *OsGLR* genes were identified in the genome, which were renamed according to their location information on chromosomes (Table 2). To characterize these putative *OsGLR* genes, we further identified their physical and chemical characteristics, including their start and stop locations, protein lengths, theoretical PI and subcellular localization. The coding sequence length of the 26 *OsGLR* genes ranged from 361 bp to 9868 bp, and the protein length ranged from 95 to 988 amino acids (Table 1). The molecular weight of the *OsGLR* genes was predicted to be between 10497.73 and 107252.1 Da (Table 1). The PI (theoretical PI) ranged from 4.05 to 9.52, with an average of 6.52 (Table 1). Subcellular localization revealed that the 26 *OsGLR* genes were located in cytoplasm, chloroplast, endoplasmic reticulum, mitochondrion, plasma membrane, vacuole and nucleus. Almost all genes were predicted to be located on the plasma membrane or chloroplast (Table 1). Different physical and chemical characteristics of the *OsGLR* genes or proteins indicated their different biological functions.

**Figure 1:**
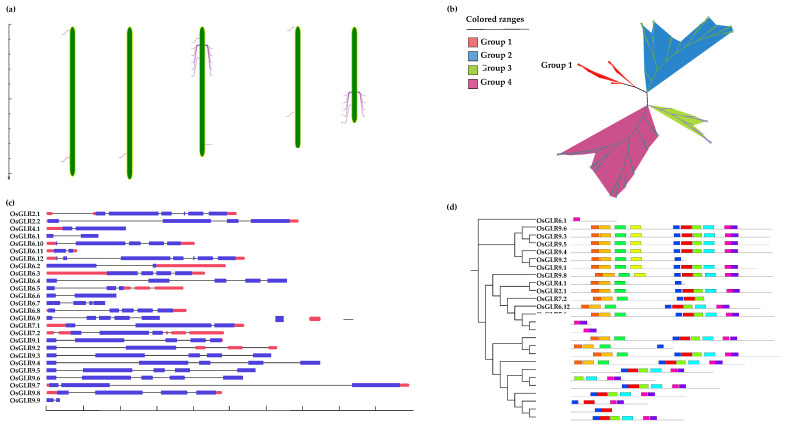
Characteristics of the *glutamate receptor-like* (*GLR*) genes in rice. (**a**) Chromosome location of the *OsGLR* genes; (**b**) phylogenetic tree of *GLR* genes in rice, tomato and Arabidopsis thaliana; (**c**) intron–exon distribution of the *OsGLR* genes; (**d**) conserved motif prediction of the *OsGLR* genes. The conserved motif can provide insights into how patterns of residue conservation and divergence in a protein family relate to functional properties, and can provide useful links to more detailed information that may be helpful in understanding that those sequences have all four subfamily/structure/function relationships. There are four subfamilies, two large subfamilies and two small subfamilies. *Arabidopsis thaliana* has only three subfamilies (groups 1, 2 and 4).

The 26 *OsGLR* genes were unevenly located on chromosomes 2, 4, 6, 7 and 9 (Figure 1a). There was only one gene on chromosomes 2 and 4, two genes on chromosomes 7 and 12, and nine genes on chromosomes 6 and 9 (Figure 1a), most of which were clustered at the end of chromosomes. To further reveal the evolution and structural characteristics of the *OsGLR* genes, we constructed a phylogenetic tree of 56 *GLR* genes using the ML (maximum likelihood) method, including 26 *GLR* genes in rice (*Oryza sativa* Japonica Group), 20 GLR genes in *A. thaliana* and 10 *GLR* genes in tomato (*Solanum lycopersicum*) (Figure 1b).

The 56 *GLR* genes were divided into two large subfamilies and two small subfamilies. The *OsGLR, AtGLR* and *SlGLR* genes were present in four, three and two subfamilies, respectively. The third subfamily was unique to the *OsGLR* genes, including eight *OsGLR* genes, namely, OsGLR6.3, OsGLR6.4, OsGLR6.5, OsGLR6.6, OsGLR6.7, OsGLR6.8, OsGLR6.9 and OsGLR6.10 (Figure 1b and Table 2), which were mostly located on chromosome 6. There were four GLR genes for rice (*OsGLR2.2, OsGLR6.1, OsGLR6.2* and *OsGLR9.7*) and *A. thaliana* (*AtGLR3.1*, *AtGLR3.2*, *AtGLR5.4* and *AtGLR5.5*) in the first subfamily. In subfamily II, there were seven *OsGLR* genes (*OsGLR9.1, OsGLR9.2, OsGLR9.3, OsGLR9.4, OsGLR9.5, OsGLR9.6* and *OsGLR9.8*), three *SIGLR* genes (*SIGLR6.1, SIGLR6.2* and *SIGLR8.1*), and nine *AtGLR* genes (*AtGLR2.2, AtGLR2.3, AtGLR2.4, AtGLR2.5. AtGLR2.6, AtGLR4.1, AtGLR5.1, AtGLR5.2* and *AtGLR5.3*). The seven *OsGLR* genes were all tightly located on chromosome 9. There were seven *GLR* genes for rice (*OsGLR2.1, OsGLR4.1, OsGLR6.11, OsGLR6.12, OsGLR7.1, OsGLR7.2* and *OsGLR9.9*), tomato (*SIGLR5.1, SIGLR7.2, SIGLR4.2, SIGLR2.1, SIGLR7.1, SIGLR2.2* and *SIGLR4.1*) and *A. thaliana* (*AtGLR2.7, AtGLR1.1, AtGLR1.2, AtGLR3.3, AtGLR4.2, AtGLR2.1* and *AtGLR2.8*) in the fourth subfamily (Figure 1b and Table 2). Previous studies have shown that the *AtGLR3.3* [20], *SIGLR3.3* [21] and *SIGLR3.5* [21] genes are associated with low temperature response, and they are located in subfamily IV.

To understand the structural characteristics of the *OsGLR* genes, the intron–exon structure of the 26 *OsGLR* genes was analyzed using the GSDS toolkit [9]. The *OsGLR* genes had 2–7 exons and 1–6 introns (Figure 1c). Different *OsGLR* genes have different structures, but it is worth noting that the genes from the same subfamily had the same or similar intron–exon structure. For example, *OsGLR9.1, OsGLR9.3, OsGLR9.4, OsGLR9.5* and *OsGLR9.6* in subfamily II had five exons and four introns. *OsGLR6.10, OsGLR6.4, OsGLR6.8* and *OsGLR6.9* in subfamily III also had five exons and four introns. O*sGLR4.1, OsGLR6.11* and *OsGLR9.9* located in subfamily IV harbored two exons and one intron (Figure 1c). We also predicted the important motifs of the *OsGLR* genes (Figure 1d and Figure S1). MEME (Multiple Em for Motif Elicitation) analysis showed that the *OsGLR* genes had 10 relatively conserved motifs ranging from 21 to 50 amino acids (Figure S1). Most of the *OsGLR* genes had ten, nine or six motifs, which was similar to the intron–exon structure. The *OsGLR* genes in the same subfamily had the same or similar motifs. *OsGLR6.3, OsGLR6.4, OsGLR6.8* and *OsGLR6.9* in subfamily III had six identical or similar motifs. *OsGLR9.3, OsGLR9.4, OsGLR9.5, OsGLR9.6* and *OsGLR9.8* had ten identical or similar motifs. *OsGLR6.12* and *OsGLR7.1* had nine identical or similar motifs. According to the phylogenetic tree, structure and motif analysis of OsGLRs, *OsGLR9.1, OsGLR9.3, OsGLR9.4, OsGLR9.5* and *OsGLR9.6* in subfamily II and *OsGLR6.4, OsGLR6.8* and *OsGLR6.9* in subfamily III had the same or similar intron–exon structure and motifs. These genes might have the same or similar biological functions, while different intron–exon structures and motifs within the same subfamily might also be involved in different functions.

### 3.2. Transmembrane (TM) Helices and Signal Peptides of OsGLR Genes

**Figure 2:**
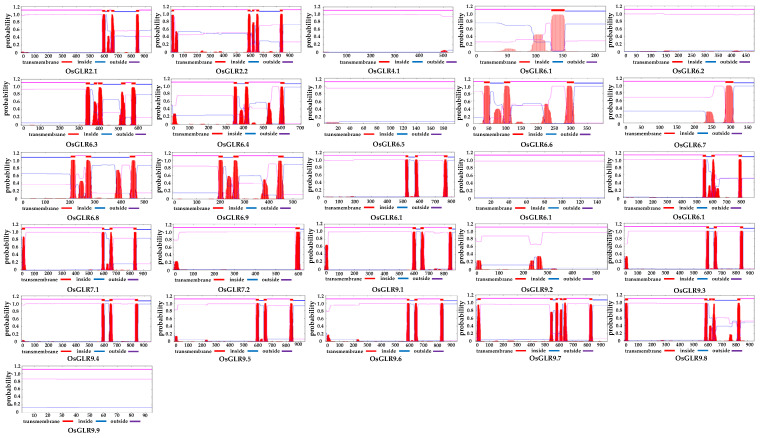
Prediction of transmembrane helices of the 26 *glutamate receptor-like* (*GLR*) genes in rice. The plot shows the posterior probabilities of inside/outside/TM helix. The plot is obtained by calculating the total probability that a residue sits in helix, inside or outside summed over all possible paths through the model.

To understand the structural characteristics of the *OsGLR* genes, we further predicted their transmembrane (TM) helices and signal peptides (Figure 2 and Figure S2). The *OsGLR* genes could be divided into two types according to TM helices: TM-related *OsGLR* genes and non-TM *OsGLR* genes. There were six genes without the TM domain (*OsGLR4.1, OsGLR6.2, OsGLR6.5, OsGLR6.11, OsGLR9.2* and *OsGLR9.8*). TM-related *OsGLR* genes could be divided into genes with one TM domain (*OsGLR6.1, OsGLR6.7* and *OsGLR7.2*), three TM domains (*OsGLR6.4, OsGLR6.6, OsGLR6.8, OsGLR6.9, OsGLR6.10, OsGLR6.12, OsGLR9.1, OsGLR9.3, OsGLR9.4, OsGLR9.5* and *OsGLR9.6*), four TM domains (*OsGLR2.1* and *OsGLR7.1*), and five TM domains (*OsGLR2.2, OsGLR6.3, OsGLR9.7* and *OsGLR9.8*). A typical glutamate receptor structure generally has four TM domains. For those *OsGLR* genes without any TM domain, they may not have all functions of glutamate receptors.

The typical GLR structure has an extracellular amino terminal domain carrying the signal peptide. The *OsGLR* genes could be divided into two types based on the presence or absence of signal peptides. Fifteen genes had signal peptides, including *OsGLR2.1, OsGLR2.2, OsGLR4.1, OsGLR6.4, OsGLR6.5, OsGLR7.1, OsGLR7.2, OsGLR9.1, OsGLR9.2, OsGLR9.3, OsGLR9.4, OsGLR9.5, OsGLR9.6, OsGLR9.7* and *OsGLR9.8*, while eleven genes had no signal peptides, including *OsGLR6.1, OsGLR6.2, OsGLR6.3, OsGLR6.6, OsGLR6.7, OsGLR6.8, OsGLR6.9, OsGLR6.10, OsGLR6.11, OsGLR6.12* and *OsGLR9.9* (Figure S2). Twelve genes contained both TM domain and signal peptide, namely *OsGLR2.1, OsGLR2.2, OsGLR6.4, OsGLR7.1, OsGLR7.2, OsGLR9.1, OsGLR9.3, OsGLR9.4, OsGLR9.5, OsGLR9.6, OsGLR9.7* and *OsGLR9.8*. These genes were very important candidate genes for further research.

### 3.3. Three-Dimensional (3D) Structure of OsGLR Genes

**Figure 3:**
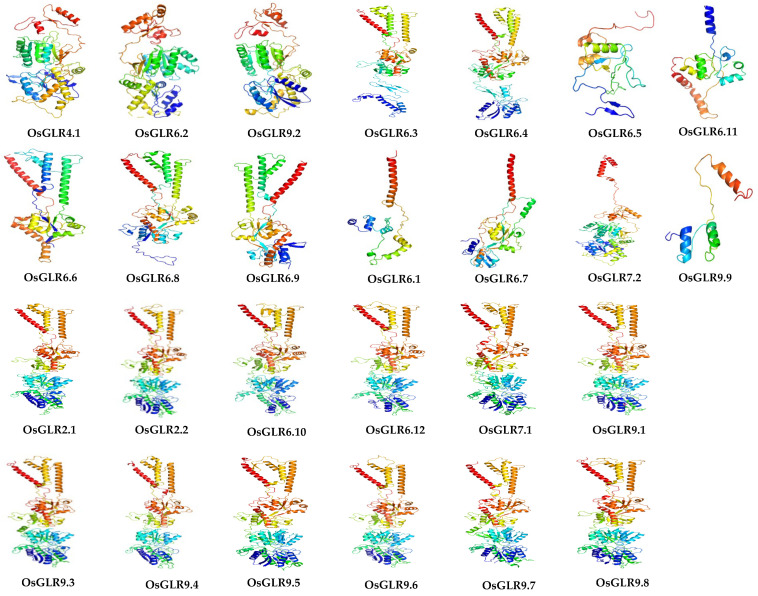
Three-dimensional (3D) structure prediction of the *glutamate receptor-like* (*GLR*) genes in rice. Green helices represent α-helices, blue arrows indicate β-strands and faint lines indicate coil. Confidence is colored from high (red) to low (blue) using a rainbow spectrum.

The 3D structure can provide important information such as sequence–structure–function relationships, interactions and active sites. By using the Phyre2 web portal [12], we could identify the distinct biological units, interactions among molecular components and secondary structures (alpha helices and beta strands) and 3D domains within individual protein molecules. The results showed that there were significant differences in the 3D structure of different *OsGLRs* (Figure 3). Interestingly, 10 *OsGLR* genes with both a TM domain and signal peptides had similar 3D structures, including *OsGLR2.1, OsGLR2.2, OsGLR7.1, OsGLR9.1, OsGLR9.3, OsGLR9.4, OsGLR9.5, OsGLR9.6, OsGLR9.7* and *OsGLR9.8* (Figure 3).

### 3.4. Cis-Acting Regulatory Elements and Functional Interaction Network of the OsGLR Genes

**Figure 4:**
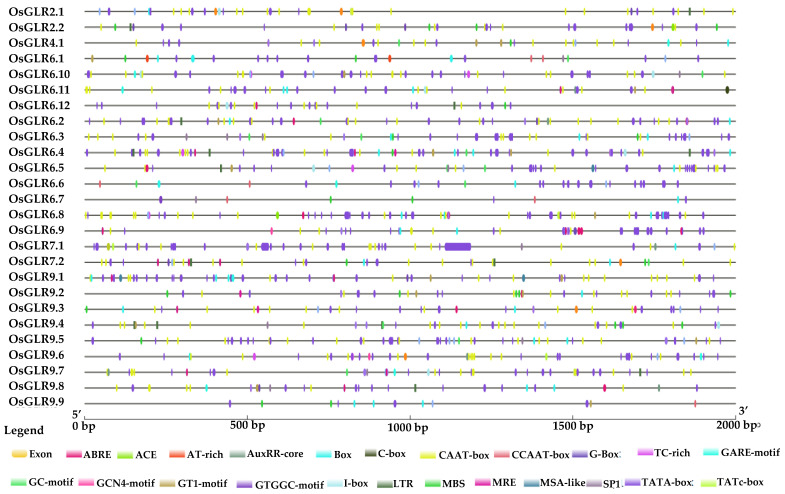
Cis-acting regulatory elements of the 26 *glutamate receptor-like* (*GLR*) genes in rice. Different colors indicate different cis-acting elements. ABRE: ABA-Responsive Element; ACE: Angiotensin-Converting Enzymes; LTR: Low-Temperature Responsiveness; MBS: Multichain Binding Site; MRE: MYB-Recognizing Elements; MSA-like: Mitosis-Specific Activator; SP1: Stress-related Protein.

To predict the function of the *OsGLR* genes, first we extracted their 2000-bp upstream promoter regions from the rice GJ genome to identify the cis-acting regulatory elements. A total of 23 cis-regulatory elements were found (Figure 4), including ABRE, ACE, AT-rich, AUXRR-core, Box, C-box, CAAT-box, G-box, TC-rich, Gare-motif, GC-motif, GCN4_motif, GT1-motif, GTGGC-motif, I-B, Ox, LTR, MBS, MRE, MSA-like, Sp1, TatA-box and TATC-Box, which could be divided into four categories, namely, light responsive elements, growth and development related elements, plant-hormone-related elements and stress-related elements. ABRE is a transcription factor, as well as the main cis-element of ABA pathway participating in drought, salt and osmotic-stress response [22,23]. The CAAT-box is mainly involved in photosynthesis [24]. The G-box plays an important role in the early senescence of rice leaves [25]. The GCN4_motif reduces protein synthesis [26]. The ten *OsGLR* genes with both the TM domain and signal peptides and similar 3D protein structures were further analyzed. The ten genes had similar structures but different cis-acting elements. The tata-box was a cis-acting regulatory element shared by all 10 of these *OsGLR* genes. Except for *OsGLR9.1*, the other nine genes all had the CAAT-box, and all genes had the G-box except for *OsGLR9.6*. *OsGLR2.1, OsGLR2.2, OsGLR7.1, OsGLR9.1, OsGLR9.3, OsGLR9.7* and *OsGLR9.8* shared the ABRE. *OsGLR2.1, OsGLR2.2, OsGLR9.4, OsGLR9.7* and *OsGLR9.8* had the LTR. *OsGLR7.1, OsGLR9.4, OsGLR9.5, OsGLR9.7* and *OsGLR9.8* all had the Sp1. *OsGLR2.1*, *OsGLR9.4* and *OsGLR9.7* commonly contained the I-box. OsGLR7.1 and OsGLR9.6 shared the TATC-box (Figure 4).

**Figure 5:**
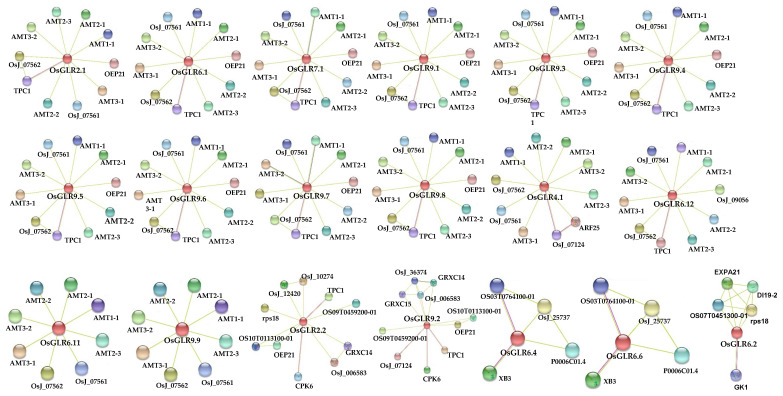
The functional interaction network of the 26 *glutamate receptor-like* (*GLR*) genes in rice. The network view summarizes the network of predicted associations for a particular group of proteins. The network nodes are proteins. The edges represent the predicted functional associations. The thickness of the lines indicates the degree of confidence prediction of the interaction. The middle red protein represents the rice glutamate receptor-like gene or the homolog of rice glutamate receptor gene. Red line—indicates the presence of fusion evidence; green line—neighborhood evidence; blue line—co-occurrence evidence; purple line—experimental evidence; yellow line—text-mining evidence; light blue line—database evidence; black line—co-expression evidence.

Then, we further predicted the functional interaction network of the *OsGLR* genes with the STRING toolkit [27]. The results showed that *OsGLR2.1, OsGLR6.1, OsGLR7.1, OsGLR9.1, OsGLR9.3, OsGLR9.4, OsGLR9.5, OsGLR9.6, OsGLR9.7* and *OsGLR9.8* had identical interacting proteins (Figure 5), including AMT3-2 (ammonium transporter 3-2), AMT3-1 (ammonium transporter 3-1), AMT2-3 (ammonium transporter 2-3), AMT2-2 (ammonium transporter 2-2), AMT2-1 (ammonium transporter 2-1), AMT1-1 (ammonium transporter 1-1), OsJ_07562 (ammonium transporter 1-2), OsJ_07561 (ammonium transporter 1-3), TPC1 (thiamine pyrophosphate carrier protein 1) and OEP21 (outer envelope pore protein 21). Among these interacting proteins, AMT3-2, AMT3-1, AMT2-3 and AMT2-2 were mainly involved in ammonium transport. OsJ_07562, OsJ_07561, AMT2-1 and AMT1-1 were transport channels for ammonium from soil. TPC1 was a two-pore calcium channel protein. OEP21 was an outer membrane pore protein. OsGLR6.4 and OsGLR6.6 had four identical interacting proteins (Figure 5), including OsJ_25737 (MAP3K-like protein), P0006C01.4 (DNA binding protein WRKY3-like), XB3 (E3 ubiquitin-protein ligase XB3) and OS03T0764100-01 (zinc finger transcription factor ZF1). OsGLR6.11 and OsGLR9.9 had eight identical interacting proteins (Figure 5), which were associated with the transport of ammonia. OsGLR4.1 and OsGLR6.12 had similar interacting proteins, while OsGLR2.2, OsGLR6.2 and OsGLR9.2 had unique interacting proteins.

Moreover, we further analyzed the ten *OsGLR* genes with both the TM domain and signal peptides and similar 3D protein structures, and they had the same interacting proteins except for OsGLR2.2. Their common interacting proteins were AMT3-2, AMT3-1, AMT2-3, AMT2-2, AMT2-1, AMT1-1, OsJ_07562, OsJ_07561, TPC1 and OEP21, which probably work through involvement in ammonium transport (Figure 5). Prediction of the cis-acting regulatory elements and interacting proteins of the *OsGLR* genes revealed that the same structure might represent the same function. However, there were sometimes exceptions. For example, OsGLR2.2 had a similar structure but different interacting proteins compared with the other nine proteins.

### 3.5. Chromatin Accessibility of OsGLR Genes

**Figure 6:**
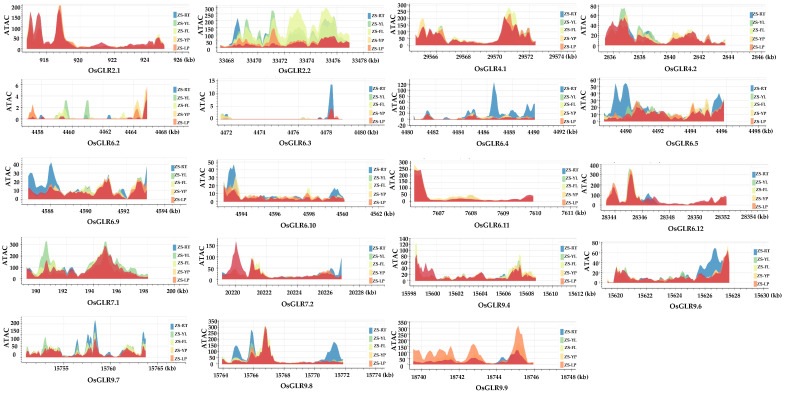
Chromatin accessibility of the *glutamate receptor-like* (*GLR*) genes in rice. Six tissues (root (RT), young leaf (YL), flag leaf (FL), young panicle (YP), lemma and palea (LP), and stamen and pistil (SP)) of Zhenshan 97 (an *indica/xian* variety) were collected for ATAC-seq experiment.

Since the *OsGLR* genes with the same structure had differences in function, we analyzed the chromatin accessibility of *OsGLR* genes to determine whether epigenetics affects their functions. We predicted the variant effects of these genes on chromatin through the RiceVarMap v2.0 toolkit [15]. The results showed that 19 *OsGLR* genes had significant differences in chromatin accessibility (Figure 6). *OsGLR6.3, OsGLR6.4, OsGLR6.5, OsGLR6.9, OsGLR6.10* and *OsGLR9.7* had the highest chromatin accessibility mainly in roots; *OsGLR2.2, OsGLR4.1* and *OsGLR9.4* exhibited the highest chromatin accessibility mainly in flag leaves; *OsGLR6.1, OsGLR6.12* and *OsGLR7.1* had the highest chromatin accessibility mainly in young leaves; and *OsGLR2.1, OsGLR7.2* and *OsGLR9.8* had the highest chromatin accessibility mainly in lemma (palea). The highest chromatin accessibility of *OsGLR6.2, OsGLR6.11, OsGLR9.6* and *OsGLR9.9* was mainly found in young ears. In particular, the chromatin accessibility of *OsGLR2.2* had a large number of peaks, which was significantly more than other *OsGLR* genes (Figure 6).

### 3.6. Evolutionary, Population Variation and Gene-Coding Sequence Haplotype (gcHap) Analysis of the GLR Genes in Rice

**Figure 7:**
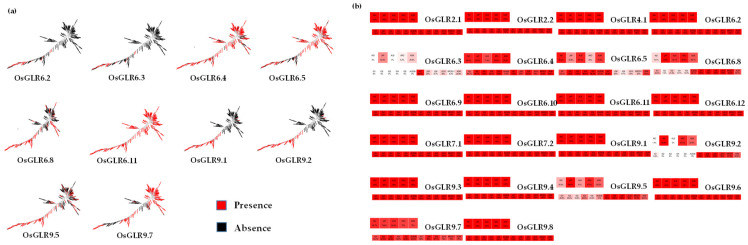
Gene presence (**a**) and distribution frequency (**b**) of the *glutamate receptor-like* (*GLR*) genes in 3010 rice accessions. The first heatmap indicates the distribution in subspecies of this gene. The second heatmap indicates the presence frequency in subgroups of this gene. A total of 3010 rice accessions were divided into five subspecies, including JAP (*japonica*, 801 accessions), IND (*indica*, 1764 accessions), AUS (*aus/boro*, 221 accessions), ARO (*aromatic basmati/sadri*, 101 accessions) and ADM (*admixed*, 123 accessions). All of these were further grouped into 12 groups according to the classification of their corresponding rice accessions. These groups include four subgroups (IG1, IG2, IG3, IG4, IG5) of *Indica* subspecies AUSG6, four subgroups (JG7, JG8, JG9, JG10) of *Japonica* subspecies AROG11, and admixtures (ADM).

Evolutionary and population variation analysis were carried out with RPAN [28] (Rice Pan-genome Browser, (Chinese Academy of Agricultural Sciences, Beijing, China)) and the RFGB [17] (Rice Functional Genomics and Breeding) database. According to the pan-genomic analysis of *OsGLR* genes, we obtained the distribution and presence frequency of the *OsGLR* genes in 453 high-quality accessions (Figure 7a,b and Figure S3). Moreover, according to the heat map of the *OsGLR* gene frequency in different subspecies and subgroups of 453 high-quality accessions, we counted the core, candidate core and distributed *OsGLR* genes. *OsGLR6.4, OsGLR6.5, OsGLR6.11* and *OsGLR9.7* were present in all 453 high-quality accessions (Figure 7a). As for the distribution of genes, the *OsGLR* genes accounted for a higher proportion in japonica subspecies (Figure 7b and Figure S3). *OsGLR2.1, OsGLR2.2, OsGLR4.1, OsGLR6.4, OsGLR6.5, OsGLR6.9, OsGLR6.10, OsGLR6.11, OsGLR6.12, OsGLR7.1, OsGLR7.2, OsGLR9.3, OsGLR9.4, OsGLR9.6, OsGLR9.7* and *OsGLR9.8* were mainly present in *japonica* subspecies, while *OsGLR6.2, OsGLR6.3, OsGLR6.8, OsGLR9.1, OsGLR9.5* and *OsGLR9.8* were mainly present in *indica* subspecies (Figure 7b and Figure S3), and were absent in other subspecies except for *OsGLR9.5*. In addition, there were twelve core genes (*OsGLR2.1, OsGLR2.2, OsGLR4.1, OsGLR6.9, OsGLR6.10, OsGLR6.12, OsGLR7.1, OsGLR7.2, OsGLR9.3, OsGLR9.4, OsGLR9.6* and *OsGLR9.8*), one candidate core gene (*OsGLR6.11*) and nine distributed genes (*OsGLR6.2, OsGLR6.3, OsGLR6.4, OsGLR6.5, OsGLR6.8, OsGLR9.1, OsGLR9.2, OsGLR9.5* and *OsGLR9.7*) in 453 high-quality accessions (Figure 7b).

**Figure 8:**
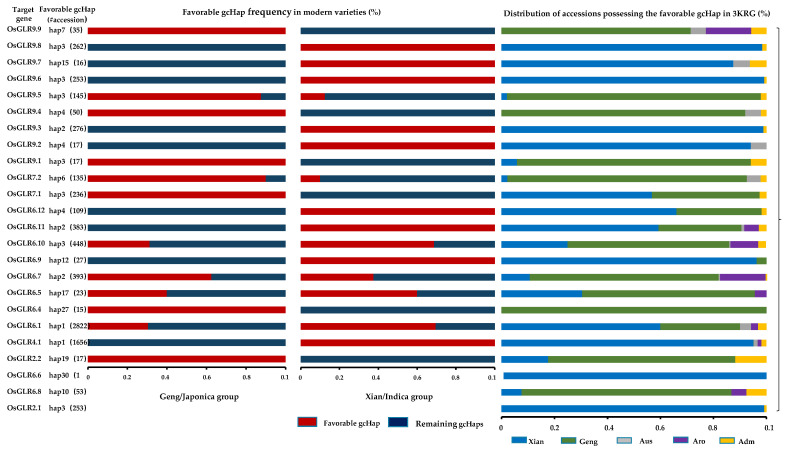
The gene-coding sequence haplotype (gcHap) diversity of the *glutamate receptor-like* (*GLR*) genes in modern and 3010 rice accessions. The 3010 rice accessions were divided into five subspecies, including *Xian* (*indica*, 1764 accessions), *Geng* (*japonica*, 801 accessions), *Aus* (*aus/boro*, 221 accessions), *Aro* (*aromatic basmati/sadri*, 101 accessions) and *Adm* (*admixed*, 123 accessions).

For gene-coding sequence haplotype (gcHap) analysis of the *OsGLR* genes (Table S1), *OsGLR6.3* and *OsGLR6.2* had no haplotype because the relevant information for these two genes could not be found in this database; therefore, gcHap analysis was performed for the 24 genes except for these 2 genes in 3010 rice genomes (3 KRG) [16]. The favorable gcHap of three genes (*OsGLR6.8*, *OsGLR2.1* and *OsGLR6.6*) was not found in modern varieties (Figure 8). Then, we analyzed the distribution of the remaining 21 genes in modern varieties and their favorable gcHap distribution in 3010 accessions (Figure 8). The favorable gcHap of many *OsGLR* genes in modern varieties showed obvious differentiation between *indica* and *japonica* (Table S1). For example, the favorable gcHap of *OsGLR9.9, OsGLR9.4, OsGLR9.1, OsGLR7.1, OsGLR6.4* and *OsGLR2.2* was mainly distributed in modern *japonica* varieties, while that of *OsGLR9.8, OsGLR9.6, OsGLR9.7, OsGLR9.2, OsGLR9.3, OsGLR6.11, OsGLR6.12, OsGLR6.9* and *OsGLR4.1* was mainly distributed in modern *indica* varieties. *Indica* varieties originated in the lower latitudes, while *japonica* varieties are more resistant to low-temperature stress than *indica* varieties. Therefore, *OsGLR9.9, OsGLR9.4, OsGLR9.1, OsGLR7.1, OsGLR6.4* and *OsGLR2.2* distributed in *japonica* varieties may be more resistant to low temperature, which is worthy of further study. Synonymous (Ks) and nonsynonymous (Ka) values could be calculated from repeated gene pairs in the rice genome. The Ka/Ks ratios of *OsGLR6.8* and *OsGLR6.9*, *OsGLR9.5* and *OsGLR9.3*, *OsGLR9.5* and *OsGLR9.6*, and *OsGLR9.6* and *OsGLR9.3* were lower than 1, indicating negative purification selection (Table 3).

### 3.7. Expression Analysis of OsGLR Genes under Multiple Abiotic Stress and Hormone Treatments

**Figure 9:**
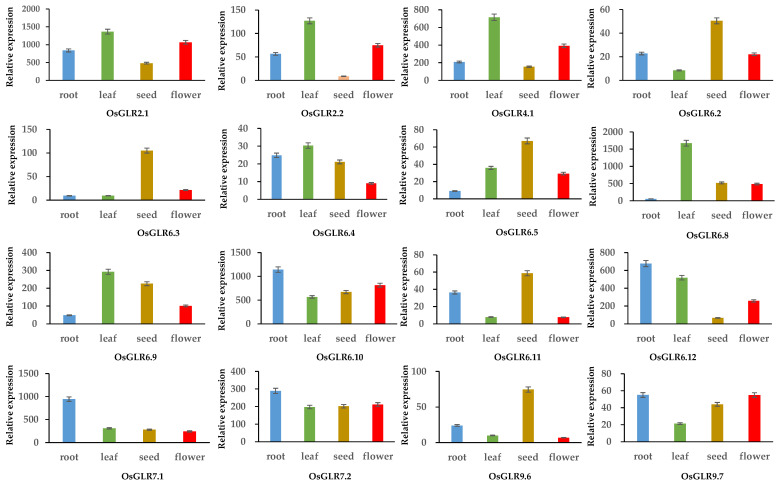
Tissue expression patterns of the *glutamate receptor-like* (*GLR*) genes in rice. Gene expression data generated by the Affymetrix ATH1 array are normalized by MAS 5.0 and RMA methods with a trimmed mean target intensity (TGT) value.

**Figure 10:**
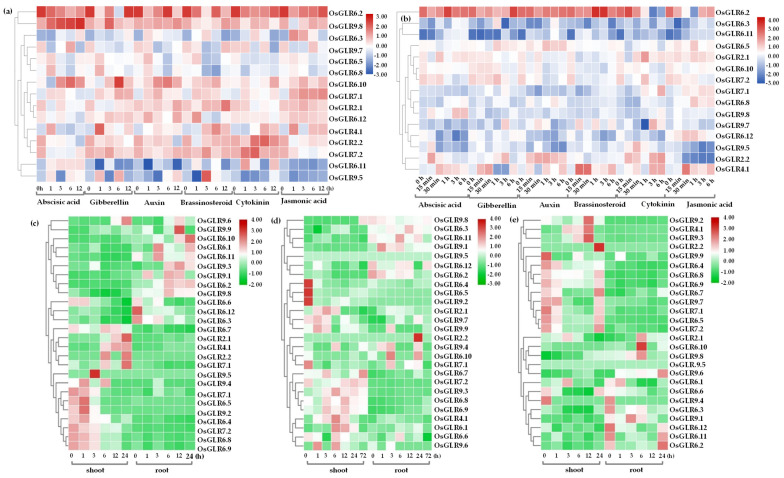
Expression analysis of the *glutamate receptor-like* (*GLR*) genes in rice. The shoot (**a**) and root (**b**) expression variation of *OsGLR* genes under a variety of hormone treatment conditions. Blue is low expression, red is high expression. Expression variation of *OsGLR* genes under drought (**c**), flood (**d**) and cold (**e**) stress conditions. Green is low expression, red is high expression.

Firstly, we analyzed the expression levels of 16 *OsGLR* genes in dry seeds, flowers, roots and young leaves (Figure 9). *OsGLR2.1, OsGLR2.2, OsGLR4.1, OsGLR6.8* and *OsGLR6.9* had the highest expression level in young leaves. The expression levels of *OsGLR6.3, OsGLR6.4, OsGLR6.11* and *OsGLR9.6* were the highest in dry seeds. *OsGLR6.10, OsGLR6.12* and *OsGLR7.1* exhibited the highest expression levels in roots (Figure 9). The expression of *OsGLR9.7* was the highest in flowers (Figure 9). The different expression levels of these genes in different tissues implied that they have different functions. In other plants, *GLR* genes are mainly involved in regulating the response to several abiotic stresses [29,30,31,32]. To identify the function of *OsGLR* genes, expression analysis of the *OsGLR* genes was carried out under multiple abiotic stress and hormone treatments (Figure 10).

Subsequently, we analyzed the expression variation of *OsGLR* genes in the roots and shoots under a variety of hormone treatments (Figure 10a,b). For the shoots, the expression of *OsGLR6.2* was down-regulated by treatment with ABA, BR, CK and JA (Figure 10a). In contrast, the expression of *OsGLR6.3* was induced by JA treatment and reached the highest level after 1 h. The expression of *OsGLR9.8* showed slow and steady increase after ABA treatment (Figure 10a). The expression of *OsGLR9.8* reached the maximum after 3 h of IAA treatment. OsGLR9.7 expression was down-regulated by IAA and GA3 treatment (Figure 10a). The expression level of *OsGLR9.7* reached the highest after 6 h of ABA treatment or JA treatment, and it was almost not detected at other indicated time points (Figure 10a). The expression of *OsGLR6.5* reached the highest level after 1 h of ABA, GA3, IAA and BR treatment. The expression of *OsGLR6.8* was the highest after 1 h of ABA, GA3 and IAA treatment (Figure 10a). The expression level of *OsGLR6.10* showed an increase immediately after IAA and BR treatment, while it decreased in the first hour and then increased to reach the highest at 6 h under ABA and GA3 treatment (Figure 10a). JA treatment up-regulated the expression of *OsGLR7.1*. The expression of *OsGLR2.1* reached the highest after 12 h of BR treatment. *OsGLR6.12* was down-regulated after CK treatment (Figure 10a). The expression of *OsGLR4.1* was up-regulated after BR and CK treatment, and that of *OsGLR2.2* decreased after ABA, IAA and GA3 treatment. *OsGLR6.11* had low expression under normal conditions and various treatments, but was up-regulated after ABA treatment. The expression level of *OsGLR9.5* was very low under various treatments and normal conditions (Figure 10a).

For the roots, *OsGLR6.2* was significantly up-regulated after 1 h of ABA treatment, 6 h of GA3 treatment, after BR treatment, 3–6 h after CK treatment, and 0.5 h after JA treatment. The expression levels of *OsGLR6.3* and *OsGLR6.11* were very low under normal conditions (Figure 10b). The expression of *OsGLR6.5*, *OsGLR6.10* and *OsGLR7.2* was limited under various hormone treatments. The expression of *OsGLR2.1* was significantly down-regulated after 15 min and up-regulated after 30 min of ABA treatment (Figure 10b). *OsGLR7.1* was significantly down-regulated after IAA treatment, but significantly up-regulated after ABA, JA and CK treatment (Figure 10b). The expression of *OsGLR6.8* was slowly but steadily up-regulated after BR treatment, and significantly up-regulated after 1 h of JA treatment. The expression of *OsGLR9.8* was significantly up-regulated after ABA treatment, but showed little change under other hormone treatments. The expression level of OsGLR9.7 decreased significantly after 1 h but increased significantly after 3 h under CK treatment. *OsGLR6.12* was down-regulated after ABA, IAA, BR and CK treatment (Figure 10b). The expression of *OsGLR9.5* was down-regulated after ABA, IAA and JA treatment. IAA and CK treatment up-regulated but JA treatment down-regulated the expression of OsGLR2.2. The expression of *OsGLR4.1* reached its highest after 30 min of ABA treatment and GA3 treatment, the lowest after 1–3 h, and the highest after 15 min of BR treatment and 30 min of JA treatment (Figure 10b).

Finally, we analyzed the expression variation of *OsGLR* genes under drought (Figure 10c), flood (Figure 10d) and cold (Figure 10d) stress conditions. Under drought stress, the expression levels of *OsGLR9.9, OsGLR6.10, OsGLR6.1, OsGLR6.11, OsGLR9.3, OsGLR9.1, OsGLR6.2, OsGLR9.8, OsGLR6.12* and *OsGLR6.3* in roots varied depending on the time of drought treatment (Figure 10c), while there was no difference in the expression of these genes in shoots. The expression level of *OsGLR9.8* was the highest in shoots after 72 h of flooding treatment, and the highest in roots without flooding treatment (Figure 10d). In shoots, *OsGLR6.4, OsGLR6.5, OsGLR9.2* and *OsGLR7.1* had the highest expression level before flooding treatment, while *OsGLR9.7, OsGLR9.9* and *OsGLR9.6* showed the highest expression level at 1 h after flooding treatment (Figure 10d), and *OsGLR9.3, OsGLR9.4, OsGLR4.1, OsGLR6.1* and *OsGLR6.6* had the highest expression at 6 h (Figure 10d). In roots, *OsGLR9.8*, *OsGLR6.12* and *OsGLR6.2* had the highest expression levels before flooding treatment, while *OsGLR9.4*, *OsGLR9.9*, *OsGLR6.10* and *OsGLR7.1* exhibited the highest expression levels at 3 h, and the expression of *OsGLR2.2* reached the highest at 24 h of flooding treatment (Figure 10d). Under low-temperature stress, there were significant differences in the expression levels of *OsGLR* genes in roots and shoots (Figure 10d). The expression of *OsGLR9.2* was the lowest at 3 h and the highest at 12 h in shoots (Figure 10d). *OsGLR4.1* and *OsGLR9.3* had the lowest expression levels in shoots before low-temperature treatment, and the highest expression at 12 h after treatment (Figure 10d). The expression level of *OsGLR2.2* in shoots remained unchanged at 12 h and before treatment, and reached the highest at 24 h (Figure 10d). However, the expression of *OsGLR9.2*, *OsGLR4.1*, *OsGLR9.3* and *OsGLR2.2* in roots showed little change. The expression levels of *OsGLR9.9, OsGLR6.4, OsGLR6.9, OsGLR7.1, OsGLR6.5, OsGLR9.4, OsGLR6.3* and *OsGLR6.12* were the highest before low-temperature treatment (Figure 10d). The expression level of *OsGLR9.9* was the highest at 1 h of low-temperature treatment. *OsGLR9.4, OsGLR6.3* and *OsGLR6.12* showed the highest expression before low-temperature treatment. The expression levels of *OsGLR6.10, OsGLR9.8, OsGLR9.5, OsGLR9.6, OsGLR9.1* and *OsGLR6.2* showed no significant change in shoots and roots before and after treatment (Figure 10d). The expression of *OsGLR6.10* and *OsGLR9.8* reached the highest at 6 h, while that of *OsGLR9.6* and *OsGLR6.2* was the highest at 24 h, and that of *OsGLR9.1* reached the highest at 3 h under low-temperature treatment (Figure 10d). 

### 3.8. Semi-Quantitative PCR Analysis of OsGLR Genes under Low-Temperature Stress

To verify whether the *OsGLR* genes in *Xian/indica* (XI) and *Geng/japonica* (GJ) are different in response to low-temperature (4℃) stress, we further performed semi-quantitative PCR of four genes (*OsGLR9.7, OsGLR9.3, OsGLR6.12* and *OsGLR4.1*) mainly distributed in Xian subspecies, and another four genes (*OsGLR9.1, OsGLR7.1, OsGLR6.4* and *OsGLR2.2*) mainly distributed in Geng subspecies (Figure S4). As a result, there were differences between these genes in expression under low-temperature stress. The four GJ *OsGLR* genes showed more obvious expression variation than the four XI OsGLR genes. For the four XI *OsGLR* genes, the expression level of OsGLR4.1 increased at 24 h and reached the highest level at 48 h (Figure S4). OsGLR9.7 had a low expression level before low-temperature stress, but its expression reached the highest level at 6 h of low-temperature stress. For OsGLR6.12 and OsGLR9.3, there was little change in expression before and after low-temperature stress. In addition, for the four GJ *OsGLR* genes, the expression level of *OsGLR6.4* was the lowest at 6 h of low-temperature treatment, and reached the highest at 48 h. *OsGLR9.1* had the highest expression before low-temperature treatment, and was almost not expressed after low-temperature treatment. The expression of *OsGLR2.2* and *OsGLR7.1* showed little difference before and after low-temperature treatment (Figure S4).

## 4. Discussion

Plant *GLR* genes are involved in numerous physiological and biochemical processes, such as signal transduction [32,33], stress response [21,30,34], growth and development [5,29,35,36,37], and stomata closure [38,39] in *A. thaliana*, tomato, radish and other species. However, this GLR gene family has not been systematically and comprehensively characterized in rice. This study for the first time systematically analyzed the *OsGLR* genes and explored their roles in the growth and development and stress response of rice.

Compared to *Arabidopsis,* rice has an additional subfamily (the third group) of *GLR* genes (Figure 1), indicating that the *GLR* genes may have more diverse and complex functions in rice. By predicting the 3D structure and interacting proteins of the *OsGLR* genes, we further analyzed the function and structure of the *OsGLR* genes. The results suggested that the genes with similar or identical protein structures may have the same function. For example, OsGLR2.1, OsGLR7.1, OsGLR9.1, OsGLR9.3, OsGLR9.4, OsGLR9.5, OsGLR9.6, OsGLR9.7 and OsGLR9.8 had similar 3D protein structures (Figure 3), and the same functional interacting proteins, including 10 proteins: AMT3-2, AMT3-1, AMT2-3, AMT2-2, AMT2-1, AMT1-1, OsJ_07562, OsJ_07561, TPC1 and OEP21 (Figure 5). These 10 interacting proteins are mainly involved in the transport and absorption of ammonium as well as two-pore calcium channel proteins and outer membrane pore proteins. The four interacting proteins of OsGLR6.4 and OsGLR6.6 were OsJ_25737, P0006C01.4, XB3 and OS03T0764100-01 (Figure 5). OsJ_25737 is a MAP3K protein and XB3 is an E3 ubiquitin protein ligase, which were both associated with low-temperature response. Therefore, OsGLR6.4 and OsGLR6.6 might be involved in low-temperature stress response.

In addition, the cis-acting elements of the 26 *OsGLR* genes could be divided into four categories, including light-responsive elements, elements related to growth and development, elements related to plant hormones, and other components (Figure 4). The light-responsive elements mainly include CAAT-box [24], ACE (angiotensin-converting enzymes), G-box, MRE (MYB-recognizing elements), and Box 4. The elements related to growth and development mainly comprise MSA-like (mitosis-specific activator) and circadian rhythm, and the elements related to plant hormone ABRE (ABA-responsive element) [23] and TATC-box [40]. There are many cis-acting elements in the promoter region of the *OsGLR* genes. The functions of some elements have been studied in other plant species, but the majority of them remain to be studied. The cis-acting element analysis of the *OsGLR* genes would help further clarify the future direction of research on this gene family. 

Notably, we found that 10 of the 26 *OsGLR* genes have the TM domain, signal peptides and similar 3D structures, including OsGLR2.1, OsGLR2.2, OsGLR7.1, OsGLR9.1, OsGLR9.3, OsGLR9.4, OsGLR9.5, OsGLR9.6, OsGLR9.7 and OsGLR9.8 (Figure 2, Figure 3 and Figure S2). Their common interacting proteins are AMT3-2, AMT3-1, AMT2-3, AMT2-2, AMT2-1, AMT1-1, OsJ_07562, OsJ_07561, TPC1 and OEP21 (Figure 5). These genes probably work by participating in ammonium transport. Moreover, the ten *OsGLR* genes had different cis-acting regulatory elements. OsGLR2.2 is structurally identical to the other nine genes but has different interacting proteins (Figure 5), which may be affected by epigenetics. We also found that the chromatin accessibility of OsGLR2.2 has a large number of peaks, which is significantly more than that of the other nine OsGLR genes (Figure 6). It can be speculated that the transcription of OsGLR2.2 is partially regulated by chromatin accessibility.

Next, in order to explore the breeding potential of OsGLR genes, we conducted population variation and gcHap analysis based on the 3K project [16,18,28]. We found that the favorable gcHap of some OsGLR genes is only present in GJ subspecies (Figure 7 and Figure S3). The favorable gcHap of many OsGLR genes in modern varieties showed obvious differentiation between *indica* and *japonica* subspecies. For example, the favorable gcHap of the six genes, *OsGLR9.9, OsGLR9.4, OsGLR9.1, OsGLR7.1, OsGLR6.4* and *OsGLR2.2*, was only found in GJ modern varieties (Figure 8). In 3010 rice accessions, the favorable gcHap of the genes *OsGLR6.2, OsGLR6.3, OsGLR6.8, OsGLR9.1, OsGLR9.2* and *OsGLR9.5* was mainly distributed in GJ subspecies (Figure 8). These genes may be more resistant to low-temperature stress. Previous research has reported that SIGLR2.1, SIGLR4.2 and AtGLR3.4 from subfamily IV are responsive to low-temperature stress by regulating apoplastic H_2_O_2_ production and redox homeostasis [21,30]. In addition, AtGLR1.2 and AtGLR1.3 in subfamily I enhance cold tolerance by increasing the endogenous JA level under cold stress [41]. Therefore, OsGLR9.9 and OsGLR7.1 in subfamily IV and OsGLR2.2 and OsGLR6.2 in subfamily I distributed in GJ subspecies are likely involved in regulating the low-temperature stress response.

Ultimately, we carried out expression analysis of the OsGLR genes under multiple abiotic stress and hormone treatments in rice (Figure 10). Under a variety of hormone treatments (ABA, GA, IAA, BR, CTK and JA), the *OsGLR* genes showed extremely complex expression variations (Figure 10a,b). Notably, the *OsGLR6.2* gene was highly expressed in root and seedling tissues under almost all hormone treatments (Figure 10a,b). We also found that the *OsGLR* genes had a strong tissue specificity of expression under drought stress (Figure 10c). For example, the expression levels of *OsGLR9.9, OsGLR6.10, OsGLR6.1, OsGLR6.11, OsGLR9.3, OsGLR9.1, OsGLR6.2, OsGLR9.8, OsGLR6.12* and *OsGLR6.3* in roots varied with the time of drought treatment (Figure 10c), while there was no significant difference in the expression of these *OsGLR* genes in shoots. To test whether the *OsGLR* genes in *indica* and *japonica* are different in response to low-temperature stress, we carried out semi-quantitative PCR of four genes mainly distributed in *indica* subspecies and another four genes mainly distributed in *japonica* subspecies (Figure S4). As a result, there were differences in expression between these genes under low-temperature stress, and those genes in *japonica* showed wider variations in expression. In short, this study carried out a systematic and comprehensive analysis of *OsGLR* genes, which has important reference value for follow-up, in-depth molecular function research.

Additionally, a recent paper found that plant GLRs participate in regeneration by dynamically altering chromatin and transcription in reprogrammed cells near callus, precisely regulating regeneration and defense [42]. Feijo and his collaborators studied the function of glutamate receptor-like proteins (GLR) in the basal land plant Physcomitrella patens, revealing a previously unknown and important role: controlling the movement of sperm in order to find the female reproductive organs and regulation of zygote development [43]. They found that this glutamate receptor-like protein functions as a channel that allows calcium ions to flow through. Many human glutamate receptors function in the same way, suggesting that the plant and human versions of this receptor maintained this conserved function during parallel evolution. We are passionate about using plants to study the function of animal neurons. Although the road ahead will be long and steep, we believe this to be a worthy pursuit.

## Figures and Tables

**Table 1:** Semi-quantitative primers of the *glutamate receptor-like* (*GLR*) genes in rice.

Gene id	Gene Name	The Upstream Primer (5’-3’)	Downstream Primers (5’-3’)
Os09t0431100	OsGLR9.7	GATGGTTGCTGATGGGGCATTC	CTTCAGGAACACCCACGTCC
Os09t0429000	OsGLR9.3	ATAACAGCTTCTGGGTATGCGAT	ATGGCTGCATCATATACCCCTAG
Os06t0680500	OsGLR6.12	CTAAACACAATCGACGAGTACGC	AATCTCTCTGGAATGCGAATCCC
Os04t0585200	OsGLR4.1	ACCGTCAATTTGTATCAGTTGATAG	ATAAGCTCCGAATAGCTTGGATTC
Os06t0188700	OsGLR6.4	TCGTGGTGGACATGACGAGC	CTTGATAAGGTCTTCGGCGGC
Os07t0103100	OsGLR7.1	ATCATCCAAGGTCTGCAGGTGAT	AGGAAGTAAGGGTATTGGGAGGA
Os02t0787600	OsGLR2.2	TGTTCGACGAGGTCATGAAGATT	CATCTGCCTTCTGTGACGACA
Os09t0428300	OsGLR9.1	AGAAAGGCAGAGGAATTCCATGT	CCAGCAAATCAAGAACTGCAGAT
Os03t0234200	OsUBQ	AAGAAGCTGAAGCATCCAGC	CCAGGACAAGATGATCTGCC

**Table 2:** Genome-wide identification and characteristics of the *glutamate receptor-like (GLR)* genes in rice.

Subfamily	RAP ID	Gene Name	No. of Amino Acids	Molecular Weight (Da)	Isoelectric Point (PI)	Aliphatic Index	Start (bp)	End (bp)	Location
I	Os02g0787600	OsGLR2.2	988	107,252.1	6.4	83.94	33,469,138	33,475,994	P(9/14),Ch(3/14)
I	Os06g0155000	OsGLR6.1	217	22,390.23	6.29	76.54	2,836,446	2,837,851	Ch(11/14),N(3/14)
I	Os06g0188400	OsGLR6.2	481	52,305.65	8.34	91.73	4,459,007	4,463,882	Cy(7/14),Ch(3/14)
I	Os09g0431100	OsGLR9.7	955	100,789.9	6.18	86.15	15,752,949	15,762,817	P(7/14),V(3/14)
II	Os09g0428300	OsGLR9.1	872	96,824.56	7.99	96.9	15,564,675	15,569,470	P(12/14)
II	Os09g0428600	OsGLR9.2	541	60,230.77	6.15	88.84	15,574,058	15,580,334	E(5/14), Ch(3/14), V(3/14)
II	Os09g0429000	OsGLR9.3	933	104,346.78	5.96	92.18	15,588,269	15,594,378	P(13/14)
II	Os09g0429200	OsGLR9.4	948	105,520.29	6.33	90.88	15,599,149	15,606,593	P(8/14),ER(3/14)
II	Os09g0429400	OsGLR9.5	934	104,377.7	5.97	91.75	15,610,715	15,616,401	P(11/14)
II	Os09g0429500	OsGLR9.6	946	105,792.31	5.94	92.32	15,620,335	15,625,678	P(13/14)
II	Os09g0431200	OsGLR9.8	950	106,149.72	5.41	90.39	15,765,945	15,770,717	P(13/14)
III	Os06g0188600	OsGLR6.3	670	74,295.45	5.87	80.9	4,472,352	4,476,661	P(8/14)
III	Os06g0188700	OsGLR6.4	702	76,300.5	9.52	81.15	4,481,240	4,487,783	P(10/14)
III	Os06g0188800	OsGLR6.5	197	21,457.38	8.45	84.67	4,489,588	4,493,909	Ch(10/14)
III	Os06g0189100	OsGLR6.6	397	43,568.45	6.08	82.92	4,500,766	4,502,665	P(6/14),V(4/14),ER(3/14)
III	Os06g0190000	OsGLR6.7	365	39,821.64	4.88	79.01	4,556,487	4,558,077	E(4/14),V(3/14)
III	Os06g0190500	OsGLR6.8	531	59,380.02	9.1	89.76	4,571,098	4,574,904	P(8/14),M(3/14)
III	Os06g0190700	OsGLR6.9	542	59,762.91	8.73	82.2	4,587,987	4,591,068	P(8/14)
III	Os06g0190800	OsGLR6.10	820	91,791.68	8.41	92.21	4,593,916	4,597,945	P(9/14), V(3/14)
IV	Os02g0117500	OsGLR2.1	944	105,303.79	5.67	94.29	918,797	923,968	P(13/14)
IV	Os04g0585200	OsGLR4.1	540	59,717.23	6.36	95.67	29,565,754	29,570,387	P(10/14)
IV	Os06g0246600	OsGLR6.11	147	15,686.37	4.05	90.27	7,607,108	7,607,939	Ch(4/14),E(5/14),M(3/14)
IV	Os06g0680500	OsGLR6.12	890	98,881.67	5.34	90.39	28,345,366	28,350,758	P(11/14)
IV	Os07g0103100	OsGLR7.1	956	104,440.94	6.22	92.01	191,053	196,429	P(10/14),ER(3/14)
IV	Os07g0522600	OsGLR7.2	637	69,593.23	5.35	93.05	20,221,077	20,225,924	P(5/14),ER(4/14),V(3/14)
IV	Os09g0485700	OsGLR9.9	95	10,497.73	4.54	83.16	18,740,826	18,741,187	P(10/14),Cy(3/14)

Note: The 26 rice glutamate receptor-like genes are divided into four subfamilies (I, II, III and IV) based on their location information on chromosomes. RAP_ID refers to the gene identifier in the Rice Annotation Project (RAP) database (https://rapdb.dna.affrc.go.jp/index.html, accessed on 29 July 2021); Gene Name is what we renamed them based on their location on the chromosome; Start and End represent the physical locations where *OsGLR* genes start and end on chromosomes; Location represents the subcellular localization of *OsGLR* genes. Cy: cytoplasmic; Ch: chloroplast thylakoid membrane; E: extracellular matrix; ER: chloroplast thylakoid membrane; M: mitochondrion; P: plasma membrane; S, sperm cell; V, vacuole; N, nucleus.

**Table 3:** KAKS analysis of the *glutamate receptor-like* (*GLR*) genes in rice.

Seq_1	Seq_2	Ka	Ks	Ka_Ks	Mya
OsGLR6.8	OsGLR6.9	0.17	0.32	0.53	24.53
OsGLR9.5	OsGLR9.3	0.06	0.27	0.22	20.93
OsGLR9.5	OsGLR9.6	0.07	0.28	0.25	21.61
OsGLR9.6	OsGLR9.3	0.06	0.27	0.22	21.07

## Data Availability

Not applicable.

## Supplementary Materials

The following supporting information can be downloaded at: https://www.mdpi.com/article/10.3390/cimb44120437/s1, Figure S1: The 10 predicted conserved motifs of the *glutamate receptor-like* (*GLR*) genes in rice. The length of 10 predicted conserved motifs ranged from 21 to 50 amino acids. Figure S2: Prediction of signal peptides and the location of their cleavage sites in the 26 glutamate receptor-like (GLR) proteins in rice. On the plot, three marginal probabilities are reported; the protein can have a Sec signal peptide (Sec/SPI), a Lipoprotein signal peptide (Sec/SPII), a Tat signal peptide (Tat/SPI) (depending on what type of signal peptide is predicted), CS (the cleavage site) or No signal peptide at all (Other, the probability that the sequence does not have any kind of signal peptide). Figure S3: Gene distribution of the *glutamate receptor-like* (*GLR*) genes in 3010 rice accessions. The 3010 rice accessions were divided into five subspecies, including JAP (*japonica*, 801 accessions), IND (*indica*, 1764 accessions), AUS (*aus/boro*, 221 accessions), ARO (*aromatic basmati/sadri*, 101 accessions) and ADM (*admixed*, 123 accessions). Figure S4: Semi-quantitative PCR analysis of the *glutamate receptor-like* (*GLR*) genes under low-temperature (4 °C) stress condition. After low-temperature stress, seeding leaves were sampled at seven different time points: 0 h, 3 h, 6 h, 9 h, 12 h, 24 h and 48 h. Table S1: Summary of favorable haplotypes of glutamate receptor genes in rice.

## References

[1] Toyota M., Spencer D., Sawai-Toyota S., Wang J.Q., Zhang T., Koo A.J., Howe G.A., Gilroy S. (2018). Glutamate triggers long-distance, calcium-based plant defense signaling. Science.

[2] Traynelis S.F., Wollmuth L.P., McBain C.J., Menniti F.S., Vance K.M., Ogden K.K., Hansen K.B., Yuan H.J., Myers S.J., Dingledine R. (2010). Glutamate Receptor Ion Channels: Structure, Regulation, and Function. Pharmacol. Rev..

[3] Chiu J.C., Brenner E.D., DeSalle R., Nitabach M.N., Holmes T.C., Coruzzi G.M. (2002). Phylogenetic and expression analysis of the glutamate-receptor-like gene family in *Arabidopsis thaliana*. Mol. Biol. Evol..

[4] Dubos C., Huggins D., Grant G.H., Knight M.R., Campbell M.M. (2003). A role for glycine in the gating of plant NMDA-like receptors. Plant J..

[5] Li J., Zhu S.H., Song X.W., Shen Y., Chen H.M., Yu J., Yi K.K., Liu Y.F., Karplus V.J., Wu P. (2006). A rice glutamate receptor-like gene is critical for the division and survival of individual cells in the root apical meristem. Plant Cell.

[6] Ni J., Yu Z.M., Du G.K., Zhang Y.Y., Taylor J.L., Shen C.J., Xu J., Liu X.Y., Wang Y.F., Wu Y.R. (2016). Heterologous Expression and Functional Analysis of Rice Glutamate Receptor-Like Family Indicates its Role in Glutamate Triggered Calcium Flux in Rice Roots. Rice.

[7] Li Y.H., Yu X.Z., Mo L.Y., Lin Y.J., Zhang Q. (2019). Involvement of glutamate receptors in regulating calcium influx in rice seedlings under Cr exposure. Ecotoxicology.

[8] Chen C.J., Chen H., Zhang Y., Thomas H.R., Frank M.H., He Y.H., Xia R. (2020). TBtools: An Integrative Toolkit Developed for Interactive Analyses of Big Biological Data. Mol. Plant.

[9] Hu B., Jin J.P., Guo A.Y., Zhang H., Luo J.C., Gao G. (2015). GSDS 2.0: An upgraded gene feature visualization server. Bioinformatics.

[10] Kumar S., Stecher G., Li M., Knyaz C., Tamura K. (2018). MEGA X: Molecular Evolutionary Genetics Analysis across Computing Platforms. Mol. Biol. Evol..

[11] Armenteros J.J.A., Tsirigos K.D., Sonderby C.K., Petersen T.N., Winther O., Brunak S., von Heijne G., Nielsen H. (2019). SignalP 5.0 improves signal peptide predictions using deep neural networks. Nat. Biotechnol..

[12] Kelley L.A., Mezulis S., Yates C.M., Wass M.N., Sternberg M.J.E. (2015). The Phyre2 web portal for protein modeling, prediction and analysis. Nat. Protoc..

[13] Mooers B.H.M. (2020). Shortcuts for faster image creation in PyMOL. Protein Sci..

[14] Bailey T.L., Elkan C. Fitting a mixture model by expectation maximization to discover motifs in biopolymers. Proceedings of the International Conference on Intelligent Systems for Molecular Biology.

[15] Zhao H., Li J.C., Yang L., Qin G., Xia C.J., Xu X.B., Su Y.M., Liu Y.M., Ming L.C., Chen L.L. (2021). An inferred functional impact map of genetic variants in rice. Mol. Plant.

[16] Wang W., Mauleon R., Hu Z., Chebotarov D., Tai S., Wu Z., Li M., Zheng T., Fuentes R.R., Zhang F. (2018). Genomic variation in 3010 diverse accessions of Asian cultivated rice. Nature.

[17] Wang C.C., Yu H., Huang J., Wang W.S., Faruquee M., Zhang F., Zhao X.Q., Fu B.Y., Chen K., Zhang H.L. (2020). Towards a deeper haplotype mining of complex traits in rice with RFGB v2.0. Plant Biotechnol. J..

[18] Zhang F., Wang C., Li M., Cui Y., Shi Y., Wu Z., Hu Z., Wang W., Xu J., Li Z. (2021). The landscape of gene-CDS-haplotype diversity in rice: Properties, population organization, footprints of domestication and breeding, and implications for genetic improvement. Mol. Plant.

[19] Zeng W., Shi J., Qiu C., Wang Y., Rehman S., Yu S., Huang S., He C., Wang W., Chen H. (2020). Identification of a genomic region controlling thermotolerance at flowering in maize using a combination of whole genomic re-sequencing and bulked segregant analysis. Theory Appl. Genet..

[20] Manzoor H., Kelloniemi J., Chiltz A., Wendehenne D., Pugin A., Poinssot B., Garcia-Brugger A. (2013). Involvement of the glutamate receptor AtGLR3.3 in plant defense signaling and resistance to Hyaloperonospora arabidopsidis. Plant J..

[21] Li H.Z., Jiang X.C., Lv X.Z., Ahammed G.J., Guo Z.X., Qi Z.Y., Yu J.Q., Zhou Y.H. (2019). Tomato GLR3.3 and GLR3.5 mediate cold acclimation-induced chilling tolerance by regulating apoplastic H_2_O_2_ production and redox homeostasis. Plant Cell Environ..

[22] Yoshida T., Fujita Y., Sayama H., Kidokoro S., Maruyama K., Mizoi J., Shinozaki K., Yamaguchi-Shinozaki K. (2010). AREB1, AREB2, and ABF3 are master transcription factors that cooperatively regulate ABRE-dependent ABA signaling involved in drought stress tolerance and require ABA for full activation. Plant J..

[23] Wang X.J., Guo C., Peng J., Li C., Wan F.F., Zhang S.M., Zhou Y.Y., Yan Y., Qi L.J., Sun K.W. (2019). ABRE-BINDING FACTORS play a role in the feedback regulation of ABA signaling by mediating rapid ABA induction of ABA co-receptor genes. New Phytol..

[24] Bezhani S., Sherameti I., Pfannschmidt T., Oelmuller R. (2001). A repressor with similarities to prokaryotic and eukaryotic DNA helicases controls the assembly of the CAAT box binding complex at a photosynthesis gene promoter. J. Biol. Chem..

[25] Liu L., Xu W., Hu X.S., Liu H.J., Lin Y.J. (2016). W-box and G-box elements play important roles in early senescence of rice flag leaf. Sci. Rep..

[26] Mittal N., Guimaraes J.C., Gross T., Schmidt A., Vina-Vilaseca A., Nedialkova D.D., Aeschimann F., Leidel S.A., Spang A., Zavolan M. (2017). The Gcn4 transcription factor reduces protein synthesis capacity and extends yeast lifespan. Nat. Commun..

[27] Szklarczyk D., Gable A.L., Nastou K.C., Lyon D., Kirsch R., Pyysalo S., Doncheva N.T., Legeay M., Fang T., Bork P. (2021). The STRING database in 2021: Customizable protein-protein networks, and functional characterization of user-uploaded gene/measurement sets. Nucleic Acids Res..

[28] Sun C., Hu Z., Zheng T., Lu K., Zhao Y., Wang W., Shi J., Wang C., Lu J., Zhang D. (2017). RPAN: Rice pan-genome browser for approximately 3000 rice genomes. Nucleic Acids Res..

[29] Ju C.L., Song Y.N., Kong D.D. (2020). Arabidopsis GLR3.5-modulated seed germination involves GA and ROS signaling. Plant Signal. Behav..

[30] Meyerhoff O., Muller K., Roelfsema M.R., Latz A., Lacombe B., Hedrich R., Dietrich P., Becker D. (2005). AtGLR3.4, a glutamate receptor channel-like gene is sensitive to touch and cold. Planta.

[31] Qi Z., Stephens N.R., Spalding E.P. (2006). Calcium entry mediated by GLR3.3, an Arabidopsis glutamate receptor with a broad agonist profile. Plant Physiol..

[32] Mousavi S.A.R., Chauvin A., Pascaud F., Kellenberger S., Farmer E.E. (2013). GLUTAMATE RECEPTOR-LIKE genes mediate leaf-to-leaf wound signalling. Nature.

[33] Grenzi M., Bonza M.C., Alfieri A., Costa A. (2021). Structural insights into long-distance signal transduction pathways mediated by plant glutamate receptor-like channels. New Phytol..

[34] Wang P.H., Lee C.E., Lin Y.S., Lee M.H., Chen P.Y., Chang H.C., Chang I.F. (2019). The Glutamate Receptor-Like Protein GLR3.7 Interacts With 14-3-3 omega and Participates in Salt Stress Response in *Arabidopsis thaliana*. Front. Plant Sci..

[35] Singh S.K., Chien C.T., Chang I.F. (2016). The Arabidopsis glutamate receptor-like gene GLR3.6 controls root development by repressing the Kip-related protein gene KRP4. J. Exp. Bot..

[36] Vincill E.D., Clarin A.E., Molenda J.N., Spalding E.P. (2013). Interacting glutamate receptor-like proteins in Phloem regulate lateral root initiation in Arabidopsis. Plant Cell.

[37] Wudick M.M., Portes M.T., Michard E., Rosas-Santiago P., Lizzio M.A., Nunes C.O., Campos C., Damineli D.S.C., Carvalho J.C., Lima P.T. (2018). CORNICHON sorting and regulation of GLR channels underlie pollen tube Ca^2+^ homeostasis. Science.

[38] Yoshida R., Mori I.C., Kamizono N., Shichiri Y., Shimatani T., Miyata F., Honda K., Iwai S. (2016). Glutamate functions in stomatal closure in Arabidopsis and fava bean. J. Plant Res..

[39] Kong D., Hu H.C., Okuma E., Lee Y., Lee H.S., Munemasa S., Cho D., Ju C., Pedoeim L., Rodriguez B. (2016). L-Met Activates Arabidopsis GLR Ca2+ Channels Upstream of ROS Production and Regulates Stomatal Movement. Cell Rep..

[40] Lv A.M., Fan N.N., Xie J.P., Yuan S.L., An Y., Zhou P. (2017). Expression of CdDHN4, a Novel YSK2-Type Dehydrin Gene from Bermudagrass, Responses to Drought Stress through the ABA-Dependent Signal Pathway. Front. Plant Sci..

[41] Zheng Y., Luo L., Wei J., Chen Q., Yang Y., Hu X., Kong X. (2018). The glutamate receptors AtGLR1.2 and AtGLR1.3 increase cold tolerance by regulating jasmonate signaling in *Arabidopsis thaliana*. Biochem. Biophys. Res. Commun..

[42] Hernández-Coronado M., Dias Araujo P.C., Ip P.L., Nunes C.O., Rahni R., Wudick M.M., Lizzio M.A., Feijó J.A., Birnbaum K.D. (2022). Plant glutamate receptors mediate a bet-hedging strategy between regeneration and defense. Dev. Cell.

[43] Ortiz-Ramírez C., Michard E., Simon A.A., Damineli D.S.C., Hernández-Coronado M., Becker J.D., Feijó J.A. (2017). Glutamate Receptor-Like channels are essential for chemotaxis and reproduction in mosses. Nature.

