# Supplementary figures and images for: Comprehensive Analysis of Glutamate Receptor-like Genes in Rice (Oryza sativa L.): Genome-Wide Identification, Characteristics, Evolution, Chromatin Accessibility, gcHap Diversity, Population Variation and Expression Analysis
Source: Curr Issues Mol Biol. 2022 Dec 16;44(12):6404–27. doi: 10.3390/cimb44120437 (PMC9777005; doi:10.3390/cimb44120437)

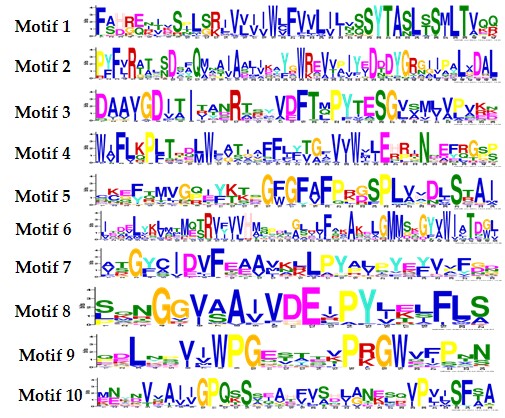

Supplement: Supplementary file 1 [file cimb-44-00437-s001.zip › Fig_S1.jpg]

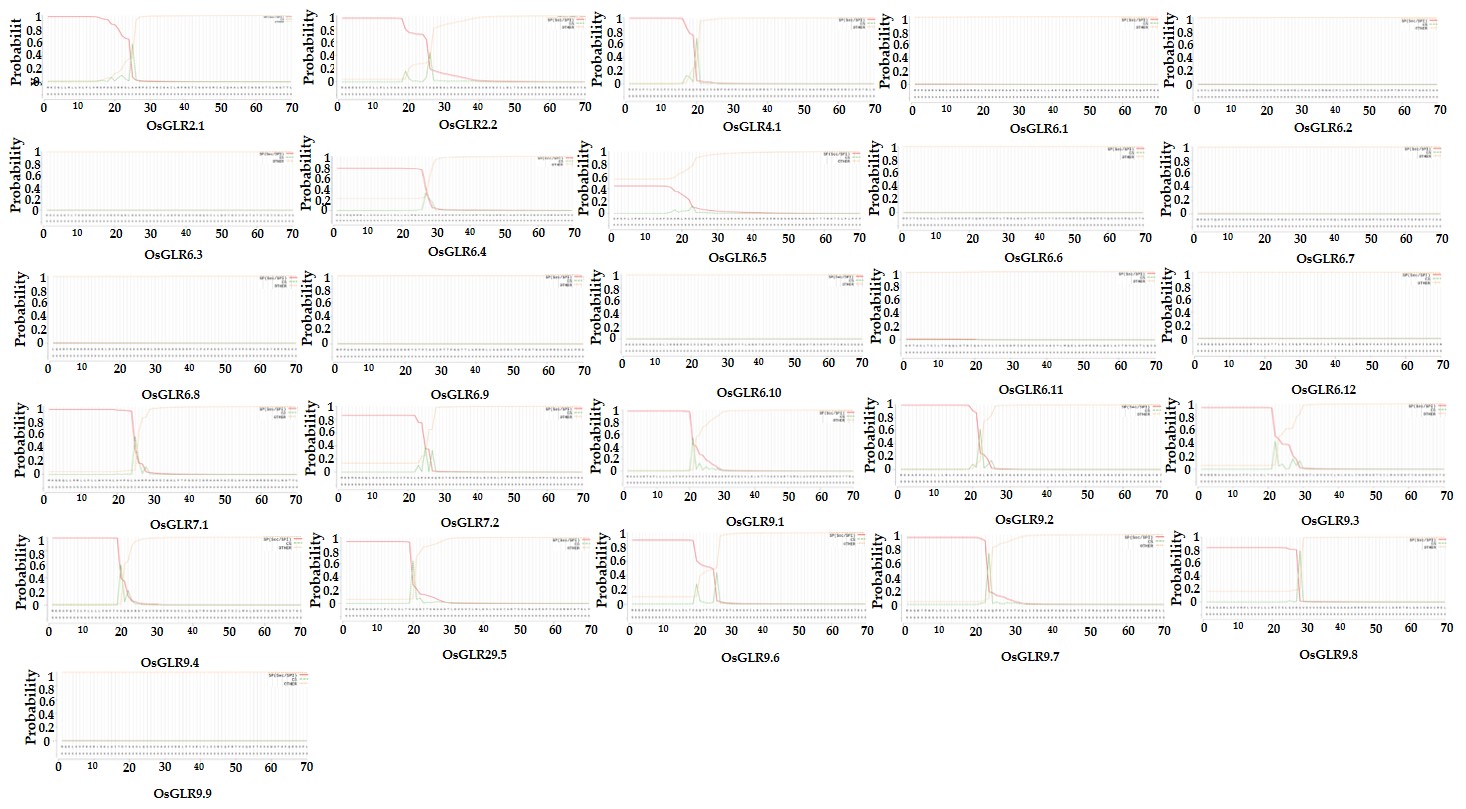

Supplement: Supplementary file 1 [file cimb-44-00437-s001.zip › Fig_S2.jpg]

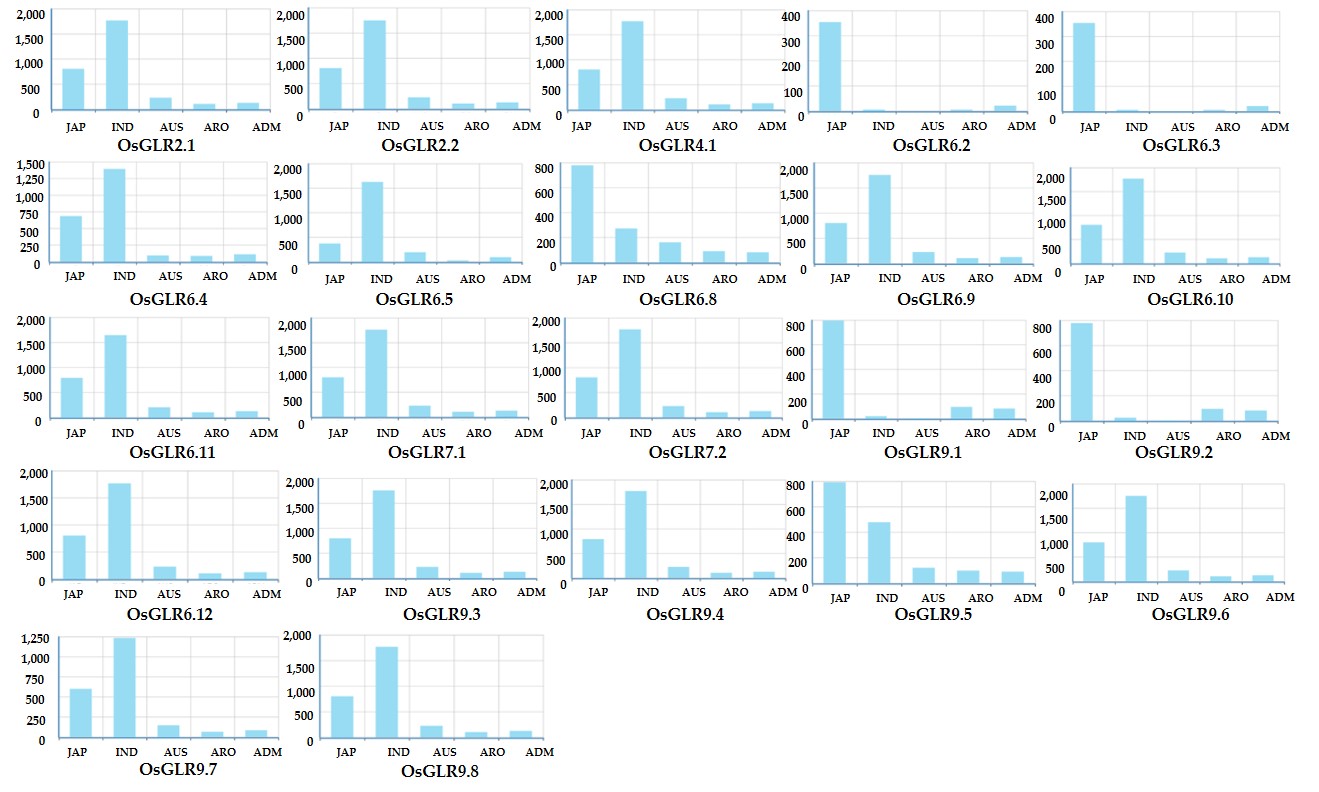

Supplement: Supplementary file 1 [file cimb-44-00437-s001.zip › Fig_S3.jpg]

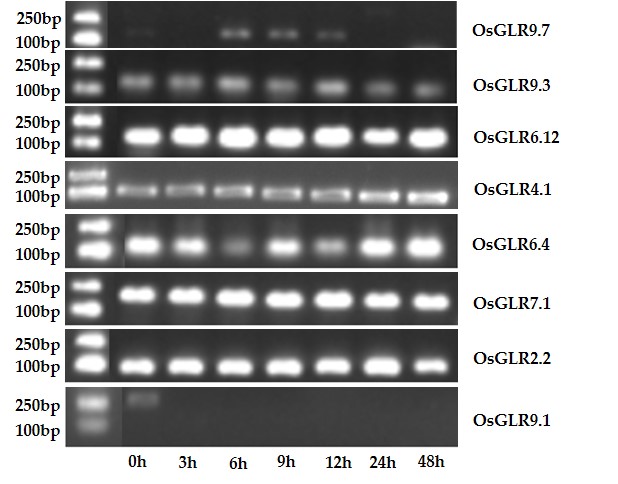

Supplement: Supplementary file 1 [file cimb-44-00437-s001.zip › Fig_S4.jpg]
